# Self‐care for coronavirus disease through electronic health technologies: A scoping review

**DOI:** 10.1002/hsr2.1122

**Published:** 2023-02-20

**Authors:** Farkhondeh Asadi, Fatemeh Rahimi, Shady Ghaderkhany, Sohrab Almasi

**Affiliations:** ^1^ Department of Health Information Technology and Management, School of Allied Medical Sciences Shahid Beheshti University of Medical Sciences Tehran Iran; ^2^ Clinical Research Development Unit, Kowsar Medical, Educational and Therapeutic Center Kurdistan University of Medical Sciences Sanandaj Iran; ^3^ Department of Health Information Technology and Management, Health Information Management, School of Allied Medical Sciences Shahid Beheshti University of Medical Sciences Tehran Iran

**Keywords:** coronavirus, electronic health, self‐care, self‐management

## Abstract

**Background and Aims:**

Considering the rapid spread and transmission of COVID‐19 and its high mortality rate, self‐care practices are of special importance during this pandemic to prevent and control the spread of the virus. In this regard, electronic health systems can play a major role in improving self‐care practices related to coronavirus disease. This study aimed to review the electronic health technologies used in each of the constituent elements of the self‐care (self‐care maintenance, self‐care monitoring, and self‐care management) during the COVID‐19 pandemic.

**Methods:**

This scoping review was conducted based on Arksey and O'Malley's framework. In this study, the specific keywords related to “electronic health,” “self‐care,” and “COVID‐19” were searched on PubMed, Web of Science, Scopus, and Google.

**Results:**

Of the 47 articles reviewed, most articles (27 articles) were about self‐care monitoring and aimed to monitor the vital signs of patients. The results showed that the use of electronic health tools mainly focuses on training in the control and prevention of coronavirus disease during this pandemic, in the field of self‐care maintenance, and medication management, communication, and consultation with healthcare providers, in the field of self‐care management. Moreover, the most commonly used electronic health technologies were mobile web applications, smart vital signs monitoring devices, and social networks, respectively.

**Conclusion:**

The study findings suggested that the use of electronic health technologies, such as mobile web applications and social networks, can effectively improve self‐care practices for coronavirus disease. In addition, such technologies can be applied by health policymakers and disease control and prevention centers to better manage the COVID‐19 pandemic.

## 1 INTRODUCTION

Coronavirus, also known as severe acute respiratory syndrome (SARS‐CoV‐2), was first identified in Wuhan City, Hubei Province, China, in December 2019.^1^ The outbreak of coronavirus was announced a pandemic by the World Health Organization (WHO) in March 2020. Despite serious global efforts to control and eradicate the virus, more than 267,865,289 laboratory approved cases and more than 5,285,888 deaths caused by this disease were recorded around the world until December 10, 2021.^2^, ^3^ The patients affected by coronavirus may exhibit no symptoms at all or some symptoms (mild to severe) within 2−14 days after exposure to the virus. In other words, any person is likely to be in exposure to asymptomatic patients. Considering the importance of coronavirus disease as a communicable infectious disease, providing self‐care services to people to fight against this disease can be considered a major achievement.^4^ As defined by WHO, self‐care refers to the ability of individuals, families, and communities to promote health, prevent disease, maintain health, and cope with illness and disability with or without the support of a healthcare provider.^5^ Self‐care is also defined as one's ability to take care of oneself and perform practices necessary to achieve, maintain or promote optimal health (including specific practices to deal with acute and chronic health problems).^6^ Based on another definition, self‐care is a process in which one takes advantage of their knowledge, skills, and abilities as a resource for taking care of their health.^7^ The three elements of this concept are self‐care maintenance, self‐care monitoring, and self‐care management.^8^

Self‐care maintenance refers to those behaviors performed to improve well‐being, preserve health, or maintain physical and emotional stability or behaviors that a patient with one or more chronic conditions performs to restore his/her health. Self‐care maintenance behaviors may be related to your lifestyle (e.g., non‐use of tobacco, having healthy foods and meals, and stress management) or medical advice (e.g., taking medicines as prescribed). Such behaviors may be also recommended by others (e.g., healthcare professionals or family members), or patients themselves decide to adopt them to achieve their health‐related goals.^8^

Self‐care monitoring is the process in which you closely observe and follow any change in your vital signs and symptoms. For example, some people regularly control their weight to achieve weight gain and loss or some people regularly visit a dentist for dental examinations to prevent or treat tooth decay. Other examples of self‐monitoring behaviors, which aim to achieve physical and emotional stability, are blood glucose checking by diabetic patients, daily weight checks by patients with heart failure, and emotional monitoring by patients with mental illnesses.^8^

Self‐care management involves the evaluation of physical and emotional changes and deciding if these changes need to be addressed. These changes may result from diseases, treatments, or the environment.^8^

Electronic health is a solution based on information and communication technology (ICT) that has attracted the attention of many researchers as a strategy to realize self‐care for the COVID‐19 pandemic.^9^, ^10^, ^11^ A variety of scientific definitions have been proposed for electronic health.^12^ WHO defines electronic health as the cost‐effective and secure use of ICT in support of health and health‐related fields, including healthcare services, health surveillance, health literature and health education, knowledge, and research. Electronic health seems to be able to benefit not only healthcare providers and health policymakers but also the general public, especially patients.^9^ According to researchers, some of the function of electronic health technologies in line with the realization of self‐care practices for coronavirus disease are as follows: access to evidence‐based information raise patients' awareness of self‐care, medication management, nutrition management,^9^, ^10^, ^11^ registration of symptoms by patients receive feedback on their health status,^9^, ^10^ recall of medical appointments, stress management, identification of high‐risk locations for COVID‐19, the introduction of specialized centers providing services related to coronavirus disease,^10^ measurement the patients' vital signs, such as blood oxygen, communication with healthcare providers,^9^ and reduction of in‐person visits to medical centers to reduce the risk of exposure to infected individuals.

Although electronic health is not a new concept in the health and medical literature, it is playing a more prominent and critical role during the COVID‐19 pandemic because of the further need of communities for such technologies.^13^ This scoping review aims to identify the electronic health technologies used in each of the constituent elements of the self‐care (self‐care maintenance, self‐care monitoring, and self‐care management) during the COVID‐19 pandemic.

## 2 METHODS

### 2.1 Design

This study employed Arksey and O'Malley's framework to conduct a scoping review.^14^ According to this framework, a scoping study consists of five main stages and one selective stage as follows: (1) Identification of the research question; (2) Identification of relevant studies; (3) Selection of studies; (4) Charting the data; (5) Collating, summarizing and reporting the results; (6) Consultation exercise. The sixth stage was not included in this review. This scoping review was also performed according to the Preferred Reporting Items for Systematic Reviews and Meta Analyses Extension for Scoping Review (PRISMA‐ScR) guidelines.^15^

### 2.2 Identification of the research question

(1) What are the applications of electronic health technologies in each of the elements of self‐care, and (2) what types of technology are used for each of the elements of self‐care?

### 2.3 Identification of relevant studies

In this study, mesh terms, and keywords related to “self‐care,” “covid‐19,” “telemedicine,” and “health informatics” were searched on PubMed, Web of Science, Scopus, and Google (Table 1). Considering the rapid changes of the COVID‐19 pandemic, online news articles such as information from official websites and health organizations in various countries were also reviewed because they were reputable and up‐to‐date sources of information on the COVID‐19 and the technologies used for self‐care of this pandemic. In Google search, the articles were selected from the studies shown on the first 10 pages of the search results; the articles presented after the first 10 pages were no longer associated with the COVID‐19 pandemic. All articles published from December 1, 2019 to November 21, 2021 were searched on the above‐mentioned databases. Updating the search strategies was conducted on December 29, 2022. To this end, two of the authors (S. A. and F. R.) searched and retrieved the articles, and any disagreements were discussed with the other three authors (F A and Sh Gh).

**Table 1:** Search strategy in PubMed database.

Search number	Query
1	(“Self Care”[Mesh] OR “self‐management”[MeSH Terms] OR “self‐care management” [Title/Abstract] OR “self‐management support” [Title/Abstract] OR “self managament” [Title/Abstract] OR “self care”[Title/Abstract] OR “self monitoring”[Title/Abstract] OR “self‐care monitoring”[Title/Abstract] OR “self‐maintenance”[Title/Abstract] OR “self‐care maintenance”[Title/Abstract] OR “symptom management”[Title/Abstract])
2	(Telemedicine[Mesh] OR “Medical Informatics”[Mesh] OR Telemedicine[Title/Abstract] OR “tele*“[All Fields] OR “digital*“[Title/Abstract] OR “remote*“[All Fields] OR “video*“[Title/Abstract] OR “Ehealth”[Title/Abstract] OR “e‐health”[Title/Abstract] OR “e‐health”[Title/Abstract] OR “electronic health”[Title/Abstract] OR mobile health[MeSH Terms] OR “mobile health”[Title/Abstract] OR “mobile app”[Title/Abstract] OR telehealth[Title/Abstract] OR Smartphone[Title/Abstract] OR “Patient Portals”[Title/Abstract] OR “social media”[Title/Abstract] OR “Internet‐Based Intervention”[Title/Abstract] OR “health information technology”[Title/Abstract])
3	(“Coronavirus”[Mesh] OR “Coronavirus”[Title/Abstract] OR “Coronavirus Infections”[Title/Abstract] OR “2019 novel coronavirus disease”[Title/Abstract] OR COVID19 OR “COVID‐19”[Title/Abstract] OR SARS‐CoV‐2[Title/Abstract] OR “SARS‐CoV‐2 infection” [Title/Abstract] OR “2019 novel coronavirus infection”[Title/Abstract] OR 2019‐nCoV infection[Title/Abstract] OR “coronavirus disease 2019”[Title/Abstract] OR “2019‐nCoV disease”[Title/Abstract])
4	#1 AND #2 AND #3

### 2.4 Selection of studies

The selection of articles included two steps: (1) title and abstract screening and (2) full‐text articles check. The authors were trained by the senior author (F. A.) to ensure the accuracy and consistency of the selection process. Then the title and abstract screening was carefully reviewed and the authors received feedback from the project manager. Two authors (S. A. and F. R.) independently evaluated the articles based on the inclusion and exclusion criteria and categorized them as “include,” “exclude,” and “undecided.” They discussed about the articles categorized as “undecided” with the senior author to take the final decision about them.

The inclusion criteria were as follows: (1) English articles and (2) articles and websites that emphasized the use of electronic health technologies regarding self‐care for coronavirus disease. The exclusion criteria were also the articles that deal with the use of electronic health technologies regarding self‐care of other diseases and 2‐articles written in languages other than English.

### 2.5 Charting the data

The data contained in the selected articles were extracted using a data extraction form, which consisted of some items, including: “publication year,” “country,” “article type” (Table 2), “concept(s) of self‐care in each study,” “relevant e‐health solutions,” and “strengths and limitations” (Table 3). Finally, the extracted data were inserted into Excel to classify and report the results. In relation to Table 3, only the articles that mentioned their strengths and limitations were added to Table.

**Table 2:** General characteristics of the included studies.

Characteristics	Number of studies (*n*)	Studies
Year of publication	2020:29	^16^, ^17^, ^18^, ^19^, ^20^, ^21^, ^22^, ^23^, ^24^, ^25^, ^26^, ^27^, ^28^, ^29^, ^30^, ^31^, ^32^, ^33^, ^34^, ^35^, ^36^, ^37^, ^38^, ^39^, ^40^, ^41^, ^42^, ^43^, ^44^
	2021:18	^45^, ^46^, ^47^, ^48^, ^49^, ^50^, ^51^, ^52^, ^53^, ^54^, ^55^, ^56^, ^57^, ^58^, ^59^, ^60^, ^61^, ^62^
	2022:8	^63^, ^64^, ^65^, ^66^, ^67^, ^68^, ^69^, ^70^
Country	South korea: 2	^16^, ^25^
	United Kingdom: 8	^17^, ^30^, ^39^, ^42^, ^53^, ^56^, ^59^, ^67^
	Italy: 3	^18^, ^22^, ^51^
	France: 2	^19^, ^23^
	USA: 6	^20^, ^26^, ^31^, ^32^, ^46^
	Finland: 1	^21^
	South Africa: 1	^24^
	Germany: 4	^27^, ^36^, ^37^, ^52^
	Greece: 1	^28^
	Poland: 1	^29^
	Netherlans: 2	^33^, ^63^
	Japan: 1	^34^
	Switzerland: 3	^35^, ^57^, ^60^
	Canada: 3	^45^, ^61^, ^69^
	Singapore: 2	^44^, ^47^
	Malaysia: 2	^38^, ^55^
	Spain: 2	^40^, ^66^
	Australia: 1	^41^
	India: 1	^43^
	Iran: 3	^49^, ^64^, ^70^
	Brazil: 1	^50^
	Denmark: 1	^54^
	Sweden: 2	^58^, ^65^
	Hong Kong: 1	^62^
	Thailand: 1	^68^
Type of publication	Journal article: 43	^16^, ^17^, ^19^, ^20^, ^21^, ^22^, ^23^, ^24^, ^25^, ^26^, ^27^, ^28^, ^29^, ^30^, ^31^, ^32^, ^33^, ^34^, ^35^, ^45^, ^48^, ^49^, ^50^, ^51^, ^52^, ^53^, ^54^, ^55^, ^56^, ^57^, ^58^, ^59^, ^60^, ^61^, ^62^
	Conference article: 1	^18^
	Website: 11	^36^, ^37^, ^38^, ^39^, ^40^, ^41^, ^42^, ^43^, ^44^, ^46^, ^47^

**Table 3:** Self‐care concepts and type of e‐health tools.

Self‐care concepts			Type of intervention	Strengths	Limitations	Studies
Self‐care maintenance	Self‐care monitoring	Self‐care management				
	Vital signs and symptoms monitoring	Communication with healthcare providers	Dashboards, cloud‐based medical image sharing, electronic prescription system a mobile app, and smart vital sign monitoring devices	Multidisciplinary approach to develop a system	‐	^16^
Psychological well‐being			Digital learning	‐	‐	^17^
Healthy lifestyle						
	Symptoms monitoring		Mobile web application	‐	‐	^18^
	Symptoms monitoring	Communication with healthcare providers	Mobile web application	‐	Lack of medical assessment and virological confirmation of COVID‐19 and comparison of application‐retrieved data with a distinct set of data, self‐checking application data could be a relevant tool to monitor outbreak trends	^19^
	Symptoms monitoring		Mobile web application	‐	‐	^20^
Self‐isolation			Social media	‐	Data collected was cross‐sectional—and hence did not account for any change over time	^21^
					Sample size	
					Unspecified content on social media	
	Symptoms monitoring	Communication with healthcare providers	Mobile web application	‐	Restrictions on use for adults and children	^22^
					Interaction and information sharing	
	Symptoms monitoring		Mobile web application	‐	‐	^23^
Psychological well‐being			Online videos	‐	Sample size	^24^
Healthy lifestyle						
	Symptoms monitoring	Communication with healthcare providers	Mobile web application	Supports multiple languages	Insufficient data	^25^
					Not providing a validation result about the algorithm	
	Symptoms monitoring		Mobile web application	‐	Availability only for adult patients and English language support	^26^
					No control group	
Motivations for social distancing			Mobile web application	‐	Not generalizability of the present results	^27^
					Cross‐sectional study	
	Symptoms monitoring	Communication with healthcare providers	Mobile web application	‐	‐	^28^
		Self‐medication	Telephone	‐	‐	^29^
	Symptoms monitoring		Mobile web application	‐	‐	^30^
	Symptoms monitoring		Mobile web application	‐	Lack of medical assessment and virological confirmation of COVID‐19 and comparison of application‐retrieved data with a distinct set of data	^31^
Social distancing	Symptoms monitoring	Communication with healthcare providers	Mobile web application	‐	The population of this study is not representative of the entire population. self‐reporting measures by the participants	^32^
Self‐isolation						
Healthy lifestyle	Symptoms monitoring		Mobile web application	‐	Interaction and information sharing	^33^
Education						
Social distancing						
Self‐isolation						
Healthy lifestyle	Symptoms monitoring		Mobile web application	‐	Restrictions on access to smartphones, tablets, or Wi‐Fi connections	^34^
			Personal health record			
Education about COVID‐19			Mobile web application	‐	Limitations in providing evidence of mHealth's impact on the quality and outcomes of patient care	^35^
Education about COVID‐19 symptoms	Symptoms monitoring		Mobile web application	‐	‐	^45^
	Symptoms monitoring		Smart technology (smartbands/smartwatches)	‐	‐	^36^
	Symptoms monitoring		Smart technology (smartbands/smartwatches)	‐	‐	^37^
	Symptoms monitoring		Mobile web application	‐	‐	^46^
	Symptoms monitoring		Mobile web application			^47^
	Symptoms monitoring		Mobile web application	‐	‐	^38^
	Symptoms monitoring		Mobile web application	‐	‐	^39^
	Symptoms monitoring		Mobile web application	‐	‐	^40^
Education about COVID‐19 symptoms	Symptoms monitoring		Mobile web application	‐	‐	^41^
Social distancing						
Self‐isolation						
Education about COVID‐19 symptoms			Mobile web application	‐	‐	^42^
Social distancing						
Self‐isolation						
Education about COVID‐19 symptoms				‐	‐	^43^
Social distancing						
Self‐isolation						
Education about COVID‐19 symptoms			Mobile web application	‐	‐	^44^
Social distancing						
Self‐isolation						
Psychological well‐being			Social media	‐	‐	^48^
Education about COVID‐19			Social media	‐	‐	^49^
Social isolation, weight changes, and physical activity			Smart technology (smartbands/smartwatches and smartphones)		Simple analysis	^50^
					Restrictions on access to smartphones, tablets, or Wi‐Fi connections	
Psychological well‐being						
Social isolation, weight changes, and physical activity			Smart technology (smartbands/smartwatches and smartphones)	‐	‐	^51^
Psychological well‐being						
Healthy lifestyle			Mobile web application	‐	Low percentage of respondent and internet access restrictions	^52^
Psychological well‐being						
Social isolation, weight changes, and physical activity			Social media	Mixed methods study	Lack of generalization due to low sample size	^53^
	Symptoms monitoring		Mobile web application	A complete description of the development process that can be replicated for the development of the future health system during a pandemic	Small number of interview	^54^
	Symptoms monitoring	Communication with healthcare providers	Mobile web application	A complete description of the development process that can be replicated for the development of the future health system during a pandemic. Using patient‐centered care approach in the development process	Not evaluating the system in the real environment	^55^
	Symptoms monitoring	Communication with healthcare providers	Home monitoring system	‐	‐	^56^
	Symptoms monitoring		Mobile web application	‐	Lack of generalization due to low sample size	^57^
Education about COVID‐19			Website	‐	Lack of generalization due to study in a country	^58^
Mental well‐being			Mobile web application	‐	Low sample size and lack of control group	^59^
Education about COVID‐19 infection prevention and control			Web based serious game	Randomization process, the triple‐blinding	Low sample size	^60^
Mental well‐being			Mobile web application	‐	Low sample size and lack of control group	^61^
Healthy lifestyle			Mobile web application		Self‐reporting measures by the participants	^62^
					Low sample size and lack of control group	
	Symptoms monitoring		Mobile web application	‐	Lack of generalization due to low sample size	^63^
	Symptoms monitoring		Mobile web application	‐	Lack of generalization due to low sample size	^64^
		Communication with healthcare providers	Mobile web application	Providing valuable insights will put in place healthcare providers, officials and scientists to guide future communications and research and support rational decision‐making in epidemic times	‐	^65^
		Emotional management	Mobile web application	‐	‐	^66^
		Communication with healthcare providers	Website	‐	Self‐reporting measures by the participants	^67^
	Symptoms monitoring	Communication with healthcare providers	Text message	‐	‐	^68^
	Symptoms monitoring		Mobile web application	‐	Lack of generalization due to low sample size	^69^
	Symptoms monitoring		Mobile web application	‐	Lack of generalization due to low sample size	^70^

### 2.6 Collating, summarizing, and reporting the results

**Figure 1:**
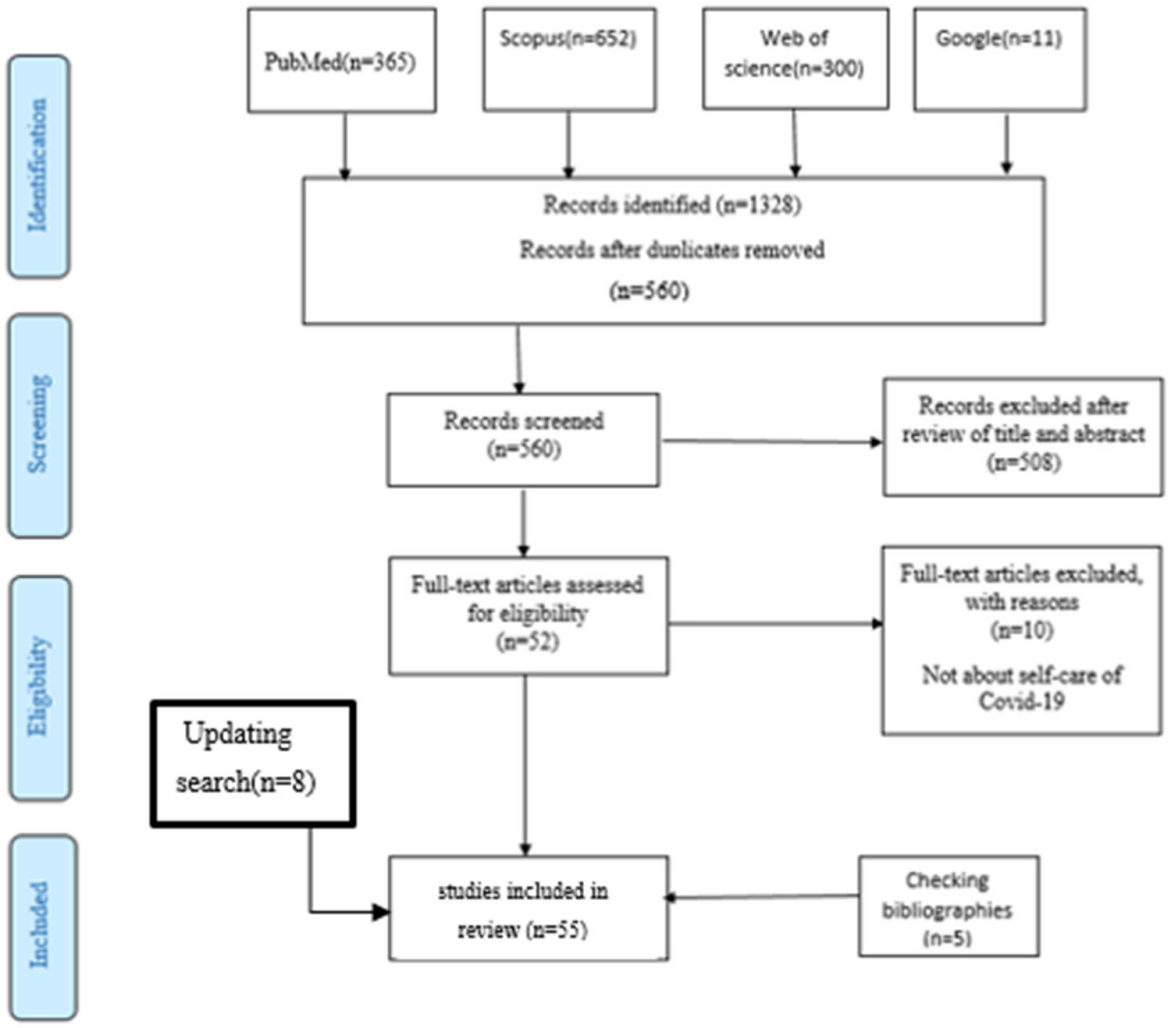
Scoping review flowchart.

At this stage, the application of electronic health technologies in each of the self‐care elements and types of the technologies used for each element were classified and reported in a table (Table 3). In addition, the geographical distribution of the studies was reported as a figure (Figure 1).

## 3 RESULTS

### 3.1 Search results

**Figure 2:**
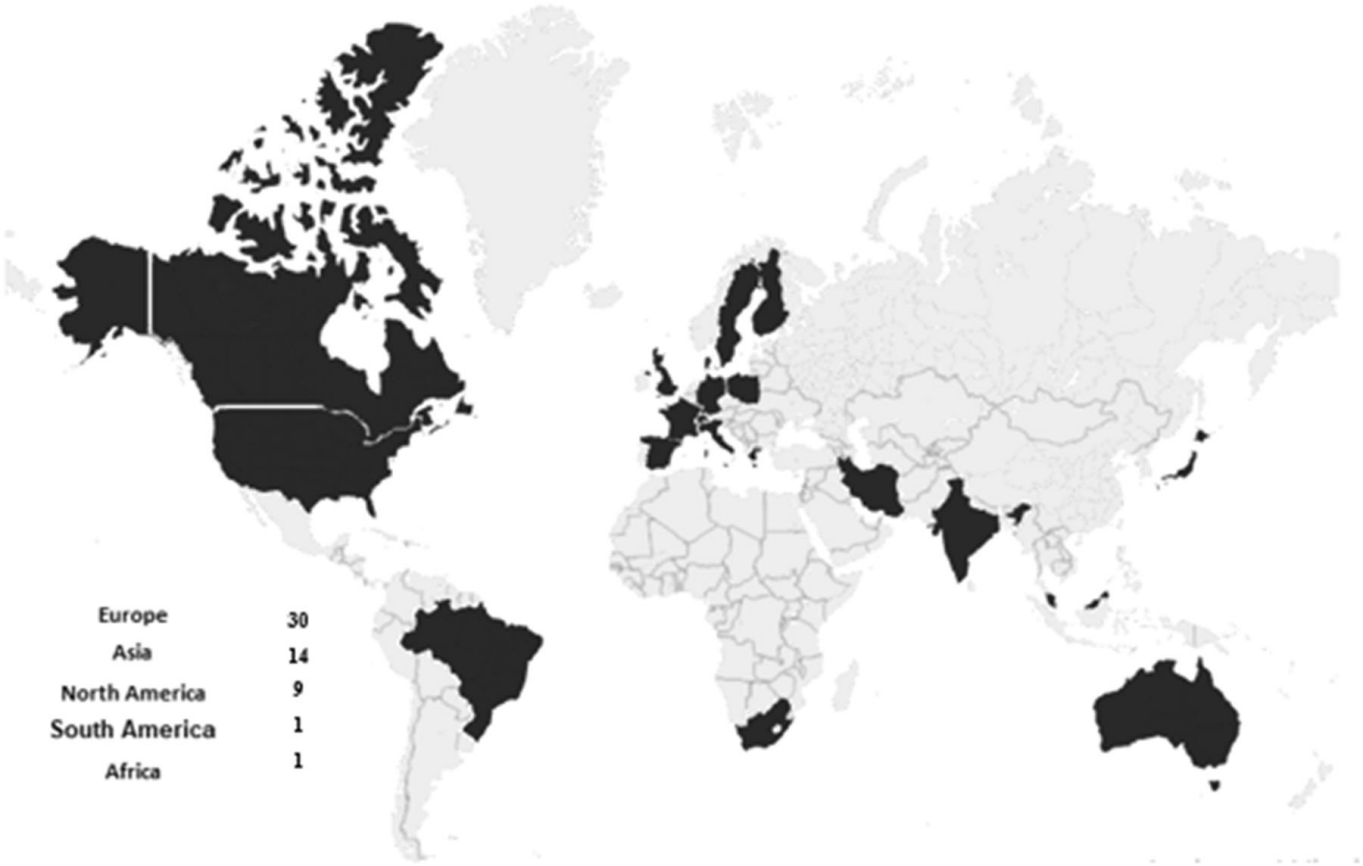
Geographical distribution of the included studies.

A total of 1328 articles were selected from the above‐mentioned databases. After eliminating duplicate articles and checking the title and abstract of the remaining articles, 52 articles were selected for further review and evaluation. When the full‐text articles were reviewed, 10 articles were eliminated because of irrelevance to self‐care for coronavirus disease. After checking the references of articles, 5 more articles were added to increase the final number of selected articles to 47. By updating the search strategy, 8 more articles were added and finally 55 articles were selected. The process of reviewing and selecting the articles is shown in Figure 2.

### 3.2 Characteristics of the studies and interventions

Based on the findings, 29 (53%), 18 (33%), and 8 (14%) studies were conducted in 2020, 2021, and 2022, respectively. In terms of geographical distribution, 30 (54%), 14 (25%), and 9 (16%) of studies were conducted in Europe, Asia, and North America, respectively. In terms of publication type, the included studies consisted of 43 (78%) journal articles, 11 (20%) articles published on websites, and 1 (2%) conference papers.

### 3.3 Self‐care concepts and type of intervention

#### 3.3.1 Self‐care maintenance

Of the 55 reviewed articles, 24 (44%) studies were related to self‐care maintenance. Using electronic health technology in this field had different objectives, including: “to manage stress and nutrition during the COVID‐19 pandemic and provide advice about prevention and control of coronavirus disease,” “to provide advice about how to use personal protective equipment,” “to provide advice about effective psychological exposure to the COVID‐19 pandemic,” “leading a healthy life during this crisis,” and “to indicate ways to control and prevent the transmission of COVID‐19.”

#### 3.3.2 Self‐care monitoring

Most studies 32 (58%) studies were about self‐care monitoring. The objective of using electronic health technologies in this field was “to monitor vital signs of patients, such as fever, cough, blood oxygen, heart rate, and respiratory rate.”

#### 3.3.3 Self‐care management

Only 12 (22%) articles dealt with the use of electronic health technologies in the field of self‐care management. These articles showed that the use of electronic health technologies in this field was mainly focused on “medication management” and “communication and consultation with healthcare providers.”

Of the 55 articles reviewed, 12 (22%) articles had emphasized at least two elements of self‐care and 43 (88%) articles focused on only one of the elements of self‐care. In terms of electronic health technologies, 37 (67%) articles had discussed the use of mobile web applications. Other technologies more commonly used in reviewed studies were smart vital sign monitoring devices 5 (9%) articles and social networks 4 (7%) articles.

## 4 DISCUSSION

### 4.1 Main finding

This scoping review aimed to identify the studies that have investigated the effects of electronic health technologies on the improvement of self‐care practices during the COVID‐19 pandemic and also to find out the elements of self‐care (self‐care maintenance, self‐care monitoring, and self‐care management) emphasized in each of these studies. The results showed that electronic health technologies used for improving self‐care practices during the COVID‐19 pandemic are mainly focused on monitoring patients' vital signs and then providing information about ways to prevent and control coronavirus disease. However, issues such as the psychological consequences of the COVID‐19 pandemic and stress management during this period have been less dealt with. Such issues are of special importance because researchers have reported that pandemic conditions increase stress, anxiety, and depression among individuals and families.^71^, ^72^, ^73^ It is hence quite clear that future self‐care interventions should pay more attention to psychological consequences and stress management during the COVID‐19 pandemic. Studies have also indicated that apps about mental health can help individuals reduce their symptoms of anxiety and depression and better manage their stress.^74^, ^75^

In terms of geographical distribution, most studies were conducted in Europe, Asia (especially East Asia), and North America. This can be attributed to the development of ICT infrastructure and the adoption of appropriate electronic health strategies for the COVID‐19 pandemic in these areas.^76^, ^77^

The study findings also indicated that the use of new technologies can facilitate the provision of services needed^78^ and equitable access to services,^79^ increase satisfaction,^80^ and reduce costs.^81^

### 4.2 Self‐care domain and e‐health used

The results demonstrated that mobile web applications were more commonly used than other electronic health technologies in reviewed studies; they were mainly applied in the field of self‐care monitoring to monitor patients' vital signs and self‐care maintenance to train people in the prevention and control of coronavirus disease. Mobile health technologies act as supportive tools in this regard. For example, mHealth is a mobile phone app that facilitates self‐care for patients with mild symptoms of coronavirus disease who have quarantined themselves at home.^82^ While emphasizing self‐care strategies for acute and chronic conditions, similar have indicated that the improved lifestyle through self‐care practices not only promotes the health status of individuals but also motivates and empowers than to participate in treatment projects.^83^ A smartphone‐based self‐care system can play an effective role in this regard by providing timely reminders and encouraging people to participate in relevant activities.^84^, ^85^, ^86^ Smartphones employ hi‐tech and provide appropriate platforms for real‐time data collection, the data that users can store and share them.^87^

Considering the pervasiveness of such technologies, the use of smartphone apps and social networks provides a good platform for preventing and training in coronavirus disease.^88^, ^89^, ^90^ A study showed that social media platforms are the source of health and well‐being information for 34% of those who seek online health information.^91^ There are a variety of smartphone apps that can be used to promote the acceptance and adoption of regular physical activity.^85^ Based on the study findings, it can be stated that the use of smartphone apps and social networks can be employed as suitable tools for distance health training and promotion of self‐care practices during the COVID‐19 pandemic. The results of this study can be also a beacon for the execution of strategies and policies adopted by the national COVID‐19 headquarters and centers for the control and prevention of coronavirus disease. These policies and strategies should consider the potential of smartphones and social networks in increasing the general public's access to health information and self‐care services during the COVID‐19 pandemic.

The results show that due to the expansion of the use of mobile applications and social networks during the epidemic, privacy and cyber security concerns should be taken into account. Future research is needed on the privacy implications of apps in this area and potential solutions to address these concerns.

Considering the high potential of using electronic health technologies in the prevention, control and management of data during the Corona epidemic, it is necessary to conduct studies for the development, establishment, and maintenance of electronic health technologies, especially mobile applications and social networks for management emerging pandemic diseases are applied at the community level.

Further large‐scale studies on the acceptance, quality, utility, usability, and effectiveness of m‐Health apps, issues related to access, security, privacy, rules, standards, emerging technologies, and infrastructures for using these tools are needed to conclusively reach the effectiveness of such digital health solutions on a large scale.

### 4.3 Strengths and limitations

#### 4.3.1 Strengths

This scoping review was conducted based on Arksey and O'Malley's framework and Systematic Reviews and the PRISMA‐ScR, which is a reputable guideline for scoping reviews. The articles were selected from among reputable databases, such as PubMed, Web of Science, Scopus, and Google, to reduce the risk of bias. Moreover, considering the rapid spread of coronavirus disease and the release of news related to the use of new technologies in the fight against this pandemic, the reputable websites that promoted the use of electronic health technologies for self‐care and preventive practices of coronavirus disease were also searched and reviewed.

#### 4.3.2 Limitations

After WHO addressed coronavirus disease as a pandemic, the rapid spread and transmission of this disease and its complications necessitated the further use of health electronic solutions and tools for dealing with this serious global health crisis. The use of electronic health technologies in different countries to fight against the COVID‐19 pandemic was mainly reflected on websites, and a few scientific articles on this subject have been published in journals. On the other hand, this study reviewed only English websites and articles and did not include websites and articles in other languages, such as Chinese, because of linguistic limitations

## 5 CONCLUSION

Considering the rapid spread of coronavirus disease and its rapid transmission and high mortality rate, the promotion of self‐care practices is of special importance for community health management during the COVID‐19 pandemic. In fact, self‐care practices can play a major role in the prevention and control of the disease and its transmission. The study findings suggested that the use of electronic health technologies, such as mobile web applications and social networks, can effectively improve self‐care practices for coronavirus disease.

## AUTHOR CONTRIBUTIONS

**Farkhondeh Asadi**: Conceptualization; supervision. **Fatemeh Rahimi**: Conceptualization; methodology; writing—original draft; writing—review & editing. **Shady Ghaderkhany**: Writing—original draft; writing—review & editing. **Sohrab Almasi**: Conceptualization; methodology; writing—original draft; writing—review & editing.

## CONFLICT OF INTEREST STATEMENT

The authors declare no conflict of interest.

## TRANSPARENCY STATEMENT

The lead author Sohrab Almasi affirms that this manuscript is an honest, accurate, and transparent account of the study being reported; that no important aspects of the study have been omitted; and that any discrepancies from the study as planned (and, if relevant, registered) have been explained.

## Data Availability

All data generated or analyzed during this study are included in this published article. All authors have read and approved the final version of the manuscript had full access to all of the data in this study and takes complete responsibility for the integrity of the data and the accuracy of the data analysis. The abstracts for these studies are available in the Web of science, Pubmed, and Scopus database.
